# DaGO-Fun: tool for Gene Ontology-based functional analysis using term information content measures

**DOI:** 10.1186/1471-2105-14-284

**Published:** 2013-09-25

**Authors:** Gaston K Mazandu, Nicola J Mulder

**Affiliations:** 1 Computational Biology Group, Department of Clinical Laboratory Sciences, Institute of Infectious Disease and Molecular Medicine, University of Cape Town, Medical School, Observatory, Cape Town, 7925, South Africa

## Abstract

**Background:**

The use of Gene Ontology (GO) data in protein analyses have largely contributed to the improved outcomes of these analyses. Several GO semantic similarity measures have been proposed in recent years and provide tools that allow the integration of biological knowledge embedded in the GO structure into different biological analyses. There is a need for a unified tool that provides the scientific community with the opportunity to explore these different GO similarity measure approaches and their biological applications.

**Results:**

We have developed DaGO-Fun, an online tool available at http://web.cbio.uct.ac.za/ITGOM, which incorporates many different GO similarity measures for exploring, analyzing and comparing GO terms and proteins within the context of GO. It uses GO data and UniProt proteins with their GO annotations as provided by the Gene Ontology Annotation (GOA) project to precompute GO term information content (IC), enabling rapid response to user queries.

**Conclusions:**

The DaGO-Fun online tool presents the advantage of integrating all the relevant IC-based GO similarity measures, including topology- and annotation-based approaches to facilitate effective exploration of these measures, thus enabling users to choose the most relevant approach for their application. Furthermore, this tool includes several biological applications related to GO semantic similarity scores, including the retrieval of genes based on their GO annotations, the clustering of functionally related genes within a set, and term enrichment analysis.

## Background

During the last decade several Gene Ontology (GO) semantic similarity approaches [1-10] have been introduced for assessing the specificity of and relationship between GO terms based on their position in the GO Directed Acyclic Graph (DAG) [11-13]. Terms in the GO DAG are semantically and topologically linked by the relations 'is_a’ and 'part_of’, expressing relations between a given child term and its parents. Semantic similarity approaches are based on these relations between terms and enable efficient exploitation of the enormous corpus of biological knowledge embedded in the GO DAG by comparing GO terms and proteins at the functional level. GO semantic similarity measures have been widely used in different contexts of protein analysis, including gene clustering, gene expression data analysis, prediction and validation of molecular interactions, and disease gene prioritization [9,14].

Initially, path- or edge-based approaches, which use a distance or the number of edges between terms in the ontology structure, were introduced [15,16]. For these approaches, the similarity score between two terms is proportional to the number of edges on the shortest path between these two terms. Path-based approaches were criticized for being limited to edge counting, ignoring positions of terms in the structure and producing uniform similarity scores [9]. Thus, information content based approaches, which rely on a numerical value to convey the description and specificity of a GO term using its position in the structure, were introduced [1]. This numerical value is called information content (IC) or semantic value, and depending on the conception of the term IC, these approaches are divided into two main families, annotation-based and topology-based families. Those depending only on the intrinsic topology of the GO structure are referred to as topology-based approaches while those using the frequencies at which terms occur in the corpus under consideration are referred to as annotation-based approaches.

Annotation-based approaches have been widely analyzed, deployed in many biological applications and were shown to outperform path-based models [17]. Most of them are adapted from Resnik [18], Lin [19] or Jiang & Conrath’s [20] methods, and are referred to as classical IC-based similarity approaches. These classical approaches use the most informative common ancestor (MICA) between terms to assess their semantic similarity. Beyond these classical approaches, several other IC-based GO semantic similarity approaches and enhancements have been suggested in order to improve annotation-based measures. These include the graph-based similarity measure (GraSM), developed by Couto et al. [7], which uses all the disjunctive common ancestors (DCA) instead of MICA, the relevance similarity approach proposed by Schlicker et al. [4], and the information coefficient idea of Li et al. [10] to correct the overestimation of similarity scores in Lin’s metric. However, the reliance of these approaches on the annotation statistics of the terms biases the scores produced [21]. Topology-based approaches, including the GO-universal metric [22], and the Zhang et al. [3] and Wang et al. [5] methods, were proposed to remove the effect of annotation dependence.

The main use of GO semantic similarity measures is the computation of protein semantic similarity or functional similarity between proteins based on their GO annotations. The completion of several genome sequencing projects has generated immense quantities of sequence data. Subsequently, with the continuous development of new high-throughput methods the amount of functional data has increased dramatically, justifying the development of dedicated methods and tools that help extract information from these data. GO [11] has successfully provided a way of consistently describing genes and proteins and a well adapted platform to computationally process data at the functional level. Protein functional similarity methods are counted among tools that allow integration of the biological knowledge contained in the GO DAG, and have contributed to the improvement of biological analyses [17]. These protein functional similarity measures have been used in several applications, including microarray data analysis [23], protein-protein interaction assessments [17], clustering and identification of functional modules in protein-protein interaction networks [24], and putative disease gene identification [25].

As well as different GO semantic similarities, several functional similarity approaches have been proposed. Some of them depend directly on the GO term IC, referred to as Direct Term- or graph-based approaches, and others are constructed via computation of GO term semantic similarity measures, referred to as Term Semantic-based approaches. The former includes approaches derived from the Jaccard, Dice and universal indices based on the Tversky ratio model of similarity [26], referred to as SimGIC [8,27], SimDIC and SimUIC [22], respectively. The latter approach includes the average (Avg) [1], best-match average (BMA) [8,22], average best matches (ABM) [5,24], and the maximum (Max) [2] combinations of GO term similarities for calculating protein functional similarities where proteins are annotated to multiple GO terms. The recent proliferation of these measures in the biomedical and bioinformatics areas was accompanied by the development of tools (http://neurolex.org/wiki/Category:Resource:Gene_Ontology_Tools) that facilitate effective exploration of these measures.

These tools include software packages and web-based online tools. Most of the software packages are implemented in the R programming language [28,29], among which we have SemSim [30], GOSim [31], and csbl.go [23]. There are also online tools, such as ProteInOn [32] and G-SESAME [33]. In addition, an integrated online tool exists, the Collaborative Evaluation of Semantic Similarity Measures (CESSM) [34], for automated evaluation of GO-based semantic similarity approaches, enabling the comparison of new measures against previously published annotation-based GO similarity measures. Evaluation is done in terms of performance with respect to sequence, Pfam and EC similarity. Note that most of the online tools do not support topology-based approaches. The G-SESAME online tool, designed by Du et al. [33] in the context of the Wang et al. approach, supports only classical Resnik [18], Jiang & Conrath [20], and Lin [19] similarity measures for protein or gene clustering applications.

The appropriate use of functional similarity measures depends on the applications [9,24] since the measures perform differently for different applications. A given measure can yield good performance for one application, but performs poorly for another. Numerous online tools have been developed, but to the best of our knowledge there is no single tool that exhaustively integrates the IC-based functional similarity metrics in order to provide researchers with the freedom to choose the most relevant approach for their specific applications. Here, this is solved through the DaGO-Fun online tool, which integrates up to 27 functional similarity measures, including topology- and annotation-based approaches. This tool also includes some important biological applications directly linked to the use of GO semantic similarity measures, namely the identification of genes based on their GO annotations, the clustering of functionally related genes within a set, and GO term enrichment analysis.

## Implementation

The DaGO-Fun tool integrates GO IC-based semantic similarity measures, allowing researchers to explore and choose an appropriate measure for their analysis. The resulting GO similarity scores are retrieved from the DaGO-Fun database implemented using MySQL and accessible via a web interface. The whole system is implemented using a LAMP (Linux-Apache-MySQL and PHP/Python) platform. This means that the DaGO-Fun tool is implemented under free software (GNU General Public Licence) using a Linux Apache server with a database structured in a relational model using MySQL, with the web interface implemented in PHP-HTML.

The back-end is composed of a set of query processing programs implemented in Python. The user input data are GO terms or UniProt proteins [35-37] and their GO annotations from the GOA project [38-41]. The database contains about 2×10^7^ proteins with GO annotations and 38 877 GO terms (25 178 biological process, 10 426 molecular function and 3 273 cellular component terms) from the GO database. The current version of DaGO-Fun uses UniProt and GOA-UniProtKB release 2013-01 of Jan 9, 2013 and GO version 1.3499 downloaded on 19-January-2013. The database will be updated using an automated scheme every three months.

### IC-based GO semantic similarity measures

We have implemented two main families of IC-based GO semantic similarity measures: annotation and topology-based families. The annotation-based methods are constrained by the annotation statistics related to terms, while topology-based measures use the intrinsic topology of the GO DAG. In terms of GO term IC, the DaGO-Fun tool includes both families and for the topology-based family, the tool implements three approaches; Zhang et al. [3], Wang et al. [5] and the GO-universal approach [22]. These topology-based family measures each has a specific scheme for computing GO term semantic similarity and functional similarity scores. The annotation-based family has been widely studied and several GO term semantic similarity and protein functional similarity approaches have been introduced.

The GO term semantic similarity approaches include traditional Resnik and Lin measures and two approaches that have been suggested to improve the performance of the Lin measure, namely Relevance (SimRel) [4] and Information Coefficient (SimIC) [10] similarity measures. Note that in the DaGO-Fun tool, the Jiang & Conrath similarity approach is under the Lin approach label as it is just the non normalized distance derived from the Lin similarity measure. Furthermore, all other normalization schemes that have been proposed have failed to improve the performance of this approach [8]. For similarity measures which are not normalized or whose values do not range between 0 and 1, we have normalized them using the uniformized information content [8,21,24], to enable users to compare these data. A value close to one indicates high similarity and close to zero indicates low similarity between proteins at the functional level.

These annotation-based GO term similarity approaches are combined using statistical measures of closeness, such as average (Avg), maximum (Max), best-match average (BMA) and averaging all the best matches (ABM) for calculating protein functional similarity scores. The difference between ABM and BMA approaches is subtle in their conception and scores produced by these two approaches differ. The ABM [5,24] for two annotated proteins is the mean of best matches of GO terms of each protein against the other, given by the following formula:

(1) $$\;\text{ABM}\left(p,q\right)\;=\;\frac{1}{n+m}\;\left(\underset{t\in T_{p}^{X}}{\sum }\underset{s\in T_{q}^{X}}{\max}S\left(s,t\right)\;+\;\underset{t\in T_{q}^{X}}{\sum }\underset{s\in T_{p}^{X}}{\max}S\left(s,t\right)\;\right)$$

The Best Match Average (BMA) [8,22] for two annotated proteins *p* and *q* is the mean of the following two values: average of best matches of GO terms annotated to protein *p* against those annotated to protein *q*, and average of best matches of GO terms annotated to protein *q* against those annotated to protein *p*, given by the following formula:

(2) $$\;\text{BMA}\left(p,q\right)\;=\;\frac{1}{2}\left(\frac{1}{n}\underset{t\in T_{p}^{X}}{\sum }\underset{s\in T_{q}^{X}}{\max}S\left(s,t\right)\;+\;\frac{1}{m}\underset{t\in T_{q}^{X}}{\sum }\underset{s\in T_{p}^{X}}{\max}S\left(s,t\right)\right)$$

In equations (1) and (2), S(s,t) is the semantic similarity score between terms *s* and *t*, $T_{r}^{X}$ is a set of GO terms in *X* representing the molecular function (MF), biological process (BP) or cellular component (CC) ontology annotating a given protein *r* and $n=\left|T_{p}^{X}\right|$ and $m=\left|T_{q}^{X}\right|$ are the number of GO terms in these sets. These two approaches produce different scores and they are equal only when *n*=*m*, which is not often the case in a set of annotated genes or proteins.

A well known issue with all these statistical measures of closeness is that they are sensitive to scores that lie at abnormal distances from the majority of scores, or outliers. This means that these measures may produce biases which affect protein functional similarity scores [22]. The functional similarity approach, SimGIC [8,27], which uses the IC of terms directly to compute protein functional similarity from their GO annotations, was introduced, and uses the Jaccard index. The DaGO-Fun tool also supports two other protein similarity measures relying on GO term IC [22]: SimDIC (Czekanowski or Lin like measure), which uses the Dice index, and SimUIC, which uses a universal index, given by the following formula:

(3) $$\text{SimDIC}\left(p,q\right)=\frac{2\times \underset{x\in A_{p}^{X}\cap A_{q}^{X}}{\sum }\text{IC}(x)}{\underset{x\in A_{p}^{X}}{\sum }\text{IC}(x)+\underset{x\in A_{q}^{X}}{\sum }\text{IC}(x)}$$

(4) $$\text{SimUIC}\left(p,q\right)=\frac{\underset{x\in A_{p}^{X}\cap A_{q}^{X}}{\sum }\text{IC}(x)}{\max\left\{\underset{x\in \overset{X}{\underset{p}{A}}}{\sum }\text{IC}(x),\underset{x\in A_{q}^{X}}{\sum }\text{IC}(x)\right\}}$$

where $A_{r}^{X}$ is a set of GO terms together with their ancestors in *X* representing the ontology (MF, BP or CC) annotating a given protein *r*. Note that these two measures are still to be evaluated and compared to the existing functional similarity measures.

The DaGO-Fun tool implements 27 functional similarity measures (see Table 1). Each of the four annotation-based GO term similarity approaches, namely Resnik, Lin, relevance and Li et al., is implemented with four known IC-based non-direct functional similarity measures (Avg, Max, BMA and ABM). DaGO-Fun also includes the three IC-based direct term functional similarity measures; SimGIC, SimDIC and SimUIC). It implements XGraSM (eXtended GraSM) in which, instead of considering only the disjunctive common ancestors (DCA), as is the case for the original GraSM, all informative common ancestors (ICA) are considered when computing semantic similarity between two different GO terms and the score between a term and itself is set to 1. This XGraSM approach has been shown to outperform the GraSM approach [21]. Note that finding the disjunctive common ancestors (DCA) between two GO terms makes the original GraSM approach computationally unattractive. Unfortunately, this computational complexity is not proportional to the improvement in performance, and thus, this approach is not included in the DaGO-Fun tool.

**Table 1:** Different GO term semantic similarity approaches and functional similarity measures implemented in DaGO-Fun

		**Functional similarity measures**							
		**Direct term-based**				**Term semantic-based**			
	**Approaches**	**SimGIC**	**SimDIC**	**SimUIC**	**SimUI**	**BMA**	**ABM**	**Avg**	**Max**
Annotation-based		x	x	x					
	XGraSM					x	x	x	x
	Resnik					x	x	x	x
	Lin					x	x	x	x
	Li et al.					x	x	x	x
	Relevance					x	x	x	x
Topology-based					x				
	Zhang et al						x		
	Wang et al.						x		
	GO-universal					x			

The letter 'x’ indicates that the relevant approach is implemented in DaGO-Fun with the corresponding functional similarity measure.

**Figure 1:**
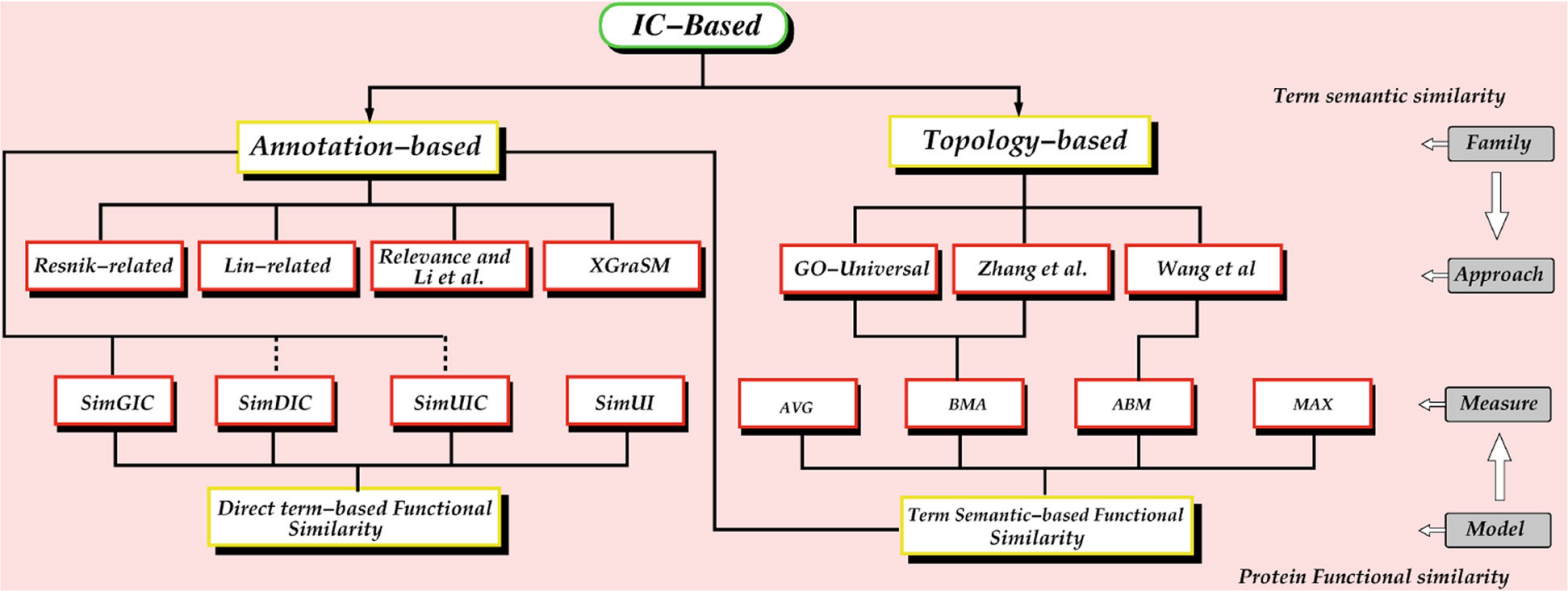
**Flowchart of all GO measures implemented in DaGO-Fun.** The solid line indicates that the performance of a given measure has already been assessed and the dashed line stands for measures or approaches that have yet to be evaluated.

On the topology-based approaches, the DaGO-Fun tool implements each approach with its associated functional similarity measure as suggested by the authors of the approach (shown in Figure 1). Thus, the GO-universal approach is implemented with the best match average (BMA) and the Wang et al. approach uses the average best matches (ABM). For the Zhang et al. approach, the DaGO-Fun tool uses averaging best matches (ABM) as it has been shown to improve the performance of this approach [24]. The SimUI approach refers to the union-intersection protein similarity measure, which is also implemented in the GOstats package of Bioconductor [31]. It is a particular case of SimGIC (using the Jaccard index) which assumes that all GO terms occur at equal frequency, in which case, only the topology of the GO DAG is needed [22].

### Retrieving information from DaGO-Fun

**Figure 2:**
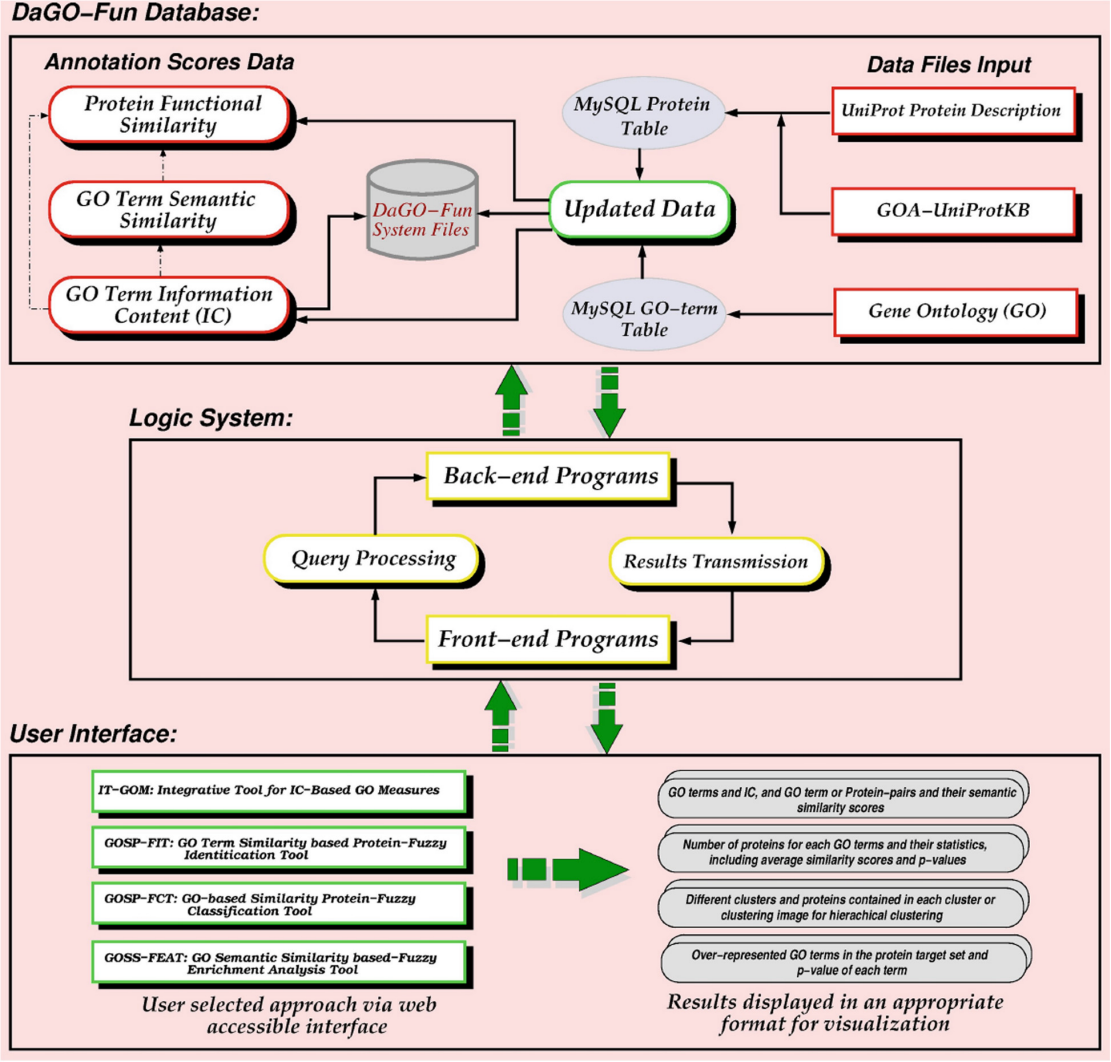
**The DaGO-Fun system architecture.** The user selects the application and enters the input (GO Ids, Protein Accessions or Gene names, GO Id pairs and protein or gene name pairs). The application is processed from the DaGO-Fun system and results are displayed in a comprehensive format for visualization.

Protein annotations were retrieved from GOA-UniProtKB at http://www.ebi.ac.uk/GOA using UniProt protein accession (ID), gene name and description. GO term topological features (term parents and level) were extracted from the GO database. These data are integrated into a MySQL database of biological concepts present in DaGO-Fun, and used to produce GO term IC, GO term semantic similarity and protein functional similarity scores. The GO term IC scores are integrated into the precompiled dictionaries in the DaGO-Fun tool. The tool is based on a client-server model and is accessible at http://web.cbio.uct.ac.za/ITGOM by any user with a standard web browser. The user interface in DaGO-Fun allows easy and comprehensive navigation, query and exploration of GO term, protein semantic similarity scores, and includes biological applications, as shown in Figure 2. This web interface allows the user to input queries in two main dynamic and customizable steps from the search to the user input options before submitting an application for processing.

#### Setting parameters step

The DaGO-Fun tool provides a comprehensive searching scheme. The user selects the task to be processed, which includes the ontology (Biological Process, Molecular Function or Cellular Component) under consideration, and chooses the GO semantic similarity measure family (annotation or topology-based). After this, he/she can select one from a list of available models, which is restricted according to the selected family. Finally, some additional options are available only when dealing with proteins, depending on the user’s choices. If the user selects the annotation-based family then more information is requested about the class (direct IC or non direct IC) of the approach selected and how the IC or GO term similarity scores should be combined. The engine changes further steps to guide the user’s choices by only making available the options relevant to the current choice.

#### User input step

After selecting appropriate parameters, the user enters their queries in a text area or from a file, and the size of the input allowed depends on the applications. Note that the DaGO-Fun tool currently includes four applications, namely: Term and protein semantic similarity measures (IT-GOM), Protein Fuzzy-Identification (GOSP-FIT), Term Fuzzy-Enrichment Analysis (GOSS-FEAT) and Protein Fuzzy-Classification (GOSP-FCT). Here, the fuzzy concept is related to the fact that the results or outputs of a given query are a function of a certain agreement score or level. 

• For IT-GOM at http://web.cbio.uct.ac.za/ITGOM/tools/itgom.php: up to 3000 pairs of GO Ids, UniProt protein accessions or gene names can be submitted for GO term similarity and functional similarity querying. For GO term IC, the user can enter up to 5000 GO Ids.

• A list of at most 20 GO Ids belonging to the same GO ontology is recommended when using GOSP-FIT at http://web.cbio.uct.ac.za/ITGOM/tools/gotspfit.php.

• For GOSS-FEAT at http://web.cbio.uct.ac.za/ITGOM/tools/gossfeat.php: a target list of at most 2000 protein UniProt accessions or gene names is recommended.

• Finally, a list of no more than 200 protein UniProt accessions or gene names is recommended for GOSP-FCT at http://web.cbio.uct.ac.za/ITGOM/tools/gospfuct.php.

These cut-offs are mainly due to the limitations of the computational resources available but also to the visualization constraints and algorithm complexity, for example when running hierarchical clustering in GOSP-FCT.

#### Outputs

**Figure 3:**
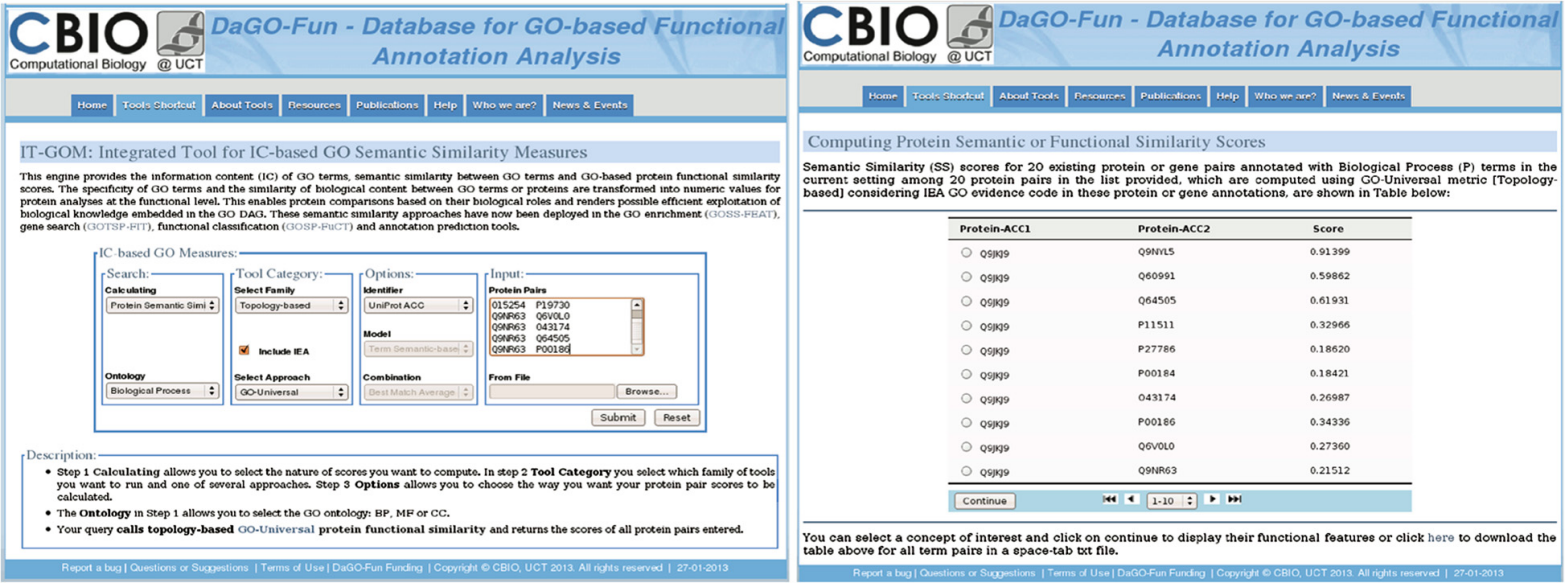
**Application example of querying IT-GOM and an output summary.** The left figure shows the DaGO-Fun interface providing the query form with user input data and the figure on the right displays the results table of protein similarity scores produced by the selected algorithm.

Comprehensive summary reports generated from the DaGO-Fun tool are made available in table format. An example of a result report is shown in Figure 3 and this report can be downloaded as a tab-delimited text file or printed. Users can query specific links directly, leading to the reported GO terms or proteins. Note that proteins are linked to their annotations via QuickGO at EBI (http://www.ebi.ac.uk/QuickGO), and for GO term semantic similarity and information content queries, GO Ids are linked to their characteristics and their sub-GO graphs displayed using AmiGO at http://amigo.geneontology.org. A given concept (protein accession or GO Id) can also be linked to more detailed results related to the concept. More details on the use of the tool are provided in the help page on the website.

### GO term statistics

The DaGO-Fun tool uses a binomial test for the retrieval of genes based of their GO annotations (GOSP-FIT) and hyper-geometric test for term enrichment analysis (GOSS-FEAT), adjusted using the Bonferroni multiple testing correction. Note that using the hyper-geometric distribution, the p-value, which is the probability of observing at least *ℓ* genes from a target gene set of size *n* by chance, knowing that the reference dataset, considered as a background distribution, contains *m* such annotated genes out of N genes is given by the following formula:

(5) PX≥ℓ=1-∑k=0ℓ-1mkN-mn-kNn

The random variable X represents the number of genes within a given target gene subset, annotated with a given GO term. Note that we are dealing with very large population size (organism’s genome, proteome or set of annotated proteins in the GOA file), in which case the size of the target gene or protein subset is very small compared to the population size. Thus, the p-value can also be approximated by or modeled using the binomial distribution [42] by taking the relative frequency of occurrence of each GO term in the reference dataset as an estimator of the probability *p* of observing the GO term under consideration. In this case, a gene taken at random from the reference dataset is an event with two possible outcomes, namely success (1), if the gene is annotated with the GO term, and failure (0) otherwise. Thus, the probability of obtaining at least *ℓ* successes in *n* trials or observing at least *ℓ* genes annotated with the GO term under consideration among *n* genes in the target set is given by the following formula:

(6) PX≥ℓ=1-∑k=0ℓ-1nkpk1-pn-k

In these cases, the lower the p-value, the less likely it is that the observed frequency of the term is due to chance, the more meaningful the term is in the target gene set. Thus, GO terms in the dataset under consideration can be ranked based on their p-values using the fact that the lower the p-value, the more significant the observed GO term is.

Note that as the biological applications implemented depend on the agreement level, the frequency of occurrence of a term through a gene or protein *g* is in fact fuzzy-frequency of this term modeled using GO similarity score $A_{g}$, of the term to the set of GO terms annotating the gene, given by the following formula:

(7) $$A_{g}(t)=S\left(t,T_{g}^{X}\right)$$

$$T_{g}^{X}$$

is a set of GO terms in the ontology *X* annotating the gene g and $S\left(t,T_{g}^{X}\right)=\max\left\{S\left(t,s\right):s\in \overset{X}{\underset{g}{T}}\right\}$[22], with S(t,s) representing the semantic similarity score between GO terms *t* and *s*. We say the gene *g* is not annotated with *t* or *t* does not occur through the gene *g* if $A_{g}(t)=0$, *g* is fully annotated with *t* or *t* fully occurs if $A_{g}(t)=1$ and *g* is fuzzy annotated with *t* or *t* fuzzy occurs if $0<A_{g}(t)<1$. Thus, the fuzzy occurrence of a given term induces the possibility of a term occurrence through a given protein in the annotation data under consideration. Specifically, the fuzzy frequency of occurrence of the GO term *t* in a set of genes C from a given experiment, denoted *ff*(*t*), is calculated using the following formula:

(8) $$\text{ff}(t)=\underset{g\in C}{\sum }\delta_{g}(t)$$

where *δ*_*g*_ is the *g*-function indicator given by

$$\delta_{g}(t)=\{\begin{array}{cc} 1 & \;\text{if}\;A_{g}(t)\geq c\\ 0 & \;\text{otherwise} \end{array}$$

*c*>0 is the agreement level or customized agreement at which the GO term *t* is considered to be a possible annotation of the gene *g*. The value of *c*=0.3 is considered to be a default value of the agreement level, and its associated fuzzy frequency is referred to as realistic or moderate frequency. This is strong or high frequency if *c*=0.7 and perfect frequency if *c*=1, which corresponds to the traditional approaches.

## Results and discussion

In this section we provide and discuss briefly some illustrations of biological applications included in the DaGO-Fun tool, namely GO Term Similarity based Protein-Fuzzy Identification Tool (GOSP-FIT), GO based Similarity Protein-Fuzzy Classification Tool (GOSP-FCT) and GO Semantic Similarity based-Fuzzy Enrichment Analysis Tool (GOSS-FEAT). We ran these applications on the *Mycobacterium tuberculosis* (MTB) genome using different GO semantic similarity approaches and analyzed the results obtained. MTB is an intracellular pathogen that causes tuberculosis (TB), one of the most threatening infectious diseases considering the severity of its impact on human populations [43]. To be successful, MTB must, at each step of the infection, express a set of genes that enables it to survive and persist inside its host macrophages, defeating antibacterial mechanisms of host cells and evading the antibiotic actions of drugs. Thus, it is believed that besides some basic biological processes, these genes or proteins must be involved in critical biological processes, such as *response to nitrosative stress* (GO:0051409), *cellular response to antibiotic* (GO:0071236), *acquisition by symbiont of nutrients from host via siderophores* (GO:0052099), *cellular lipid metabolic process* (GO:0044255), etc. We used these GO biological process terms as initial data or input for running different biological applications in the DaGO-Fun tool at moderate agreement, unless otherwise stated.

### Performing DaGO-Fun applications

Using the biological process terms listed above, we ran GOSP-FIT to identify proteins involved in a process similar to the input processes, using the GO-universal metric, Wang et al., Zhang et al, Resnik, Lin and Lin with Li et al. enhancement similarity measures. Results are shown in Table 2. We see that, except for GO-universal and Resnik approaches, other approaches tend to select more proteins for a given term. This is an indication that these approaches are overestimating GO term similarity scores. It is already known that the Lin approach overestimates similarity scores between terms, which is why the enhancement of this measure has been suggested through the information coefficient idea of Li et al. [10] and the relevance similarity approach proposed by Schlicker et al. [4] to correct these overestimated scores. From the number of proteins detected by Lin and its enhancement proposed by Li et al., we observe that this enhancement is trying to reduce the impact of Lin similarity score overestimation even though overall these measures are still overestimating similarity scores. Finally, note that one can display all proteins identified for a given term by selecting the row of the term and clicking on the 'Continue’ button.

**Table 2:** Results obtained after running the GOSP-FIT for specific GO Ids and using different GO term semantic similarity approaches

			**Number of proteins detected**							
**GO ID**	**Level**	**GO name**	**GA**	**WA**	**ZA**	**RA**	**LA**	**LLA**	**p-value**	**Corrected p-value**
GO:0044255	4	Cellular lipid metabolic process	154	1590	1907	225	1652	1286	0.00e+00	0.00e+00
GO:0071236	6	Cellular response to antibiotic	123	307	545	277	739	588	2.42e-14	9.68e-14
GO:0051409	3	Response to nitrosative stress	91	226	426	243	435	418	1.07e-14	4.26e-14
GO:0052099	6	Acquisition by symbiont of nutrients	47	128	463	2	418	269	2.75e-14	1.10e-13
		from host via siderophores								

GO-universal (GA), Wang et al. (WA), Zhang et al. (ZA), Resnik (RA), Lin (LA) and Li et al. (LLA).

**Figure 4:**
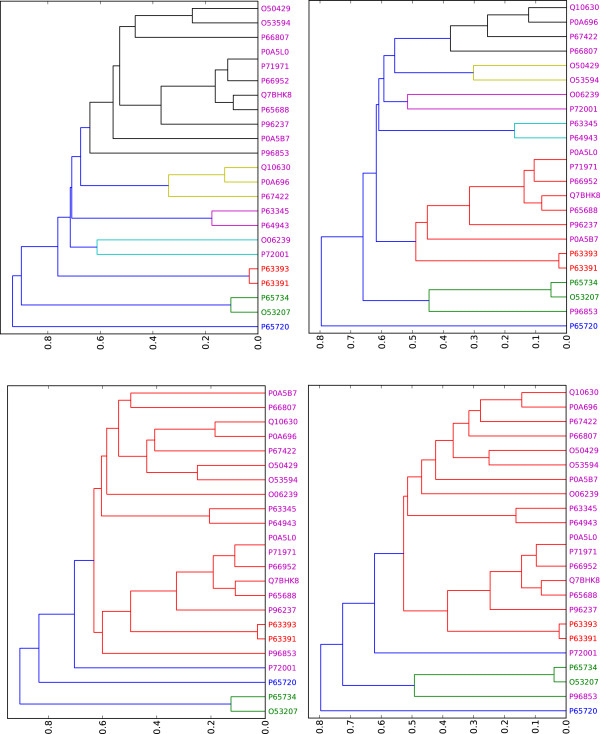
**Clustering results obtained by running the hierarchical clustering program.** Clustering results obtained by running the hierarchical clustering program using different similarity metrics under the DaGO-Fun tool. Protein label is colored according to the process in which the protein is involved. Magenta for proteins involved in GO:00051409, blue for GO:0071236, green for GO:0044255 and red for GO:0052099. **(1)** Using GO-universal approach. **(2)** Using Wang et al. approach. **(3)** Using Resnik approach. **(4)** Using Li et al. approach.

Before running other applications, we first identified in the MTB genome all genes or proteins involved in the GO annotations under consideration. A total of 23 proteins have been identified with 18 proteins (O53594, P66807, P0A696, P0A5L0, Q10630, P72001, P96853, O06239, P65688, P64943, O50429, P66952, P63345, P96237, P67422, Q7BHK8, P0A5B7, P71971) for GO:00051409, one protein (P65720) for GO:0071236, 2 (P65734, O53207) for GO:0044255, and 2 (P63391, P63393) for GO:0052099. We used these proteins as input data for running GOSP-FCT using hierarchical clustering under the customized agreement level. Results are depicted in Figure 4 and indicate that the clustering outcome depends strongly on the similarity approach used. Here, again we see that the GO-universal approach performs better than other approaches, producing a clustering image which is consistent with mapping between GO terms and identified proteins, as indicated above. It is worth mentioning that two other clustering approaches are implemented under the DaGO-Fun tool, namely the graph spectral or kmeans clustering approach and the community detecting model [44], which is referred to as a model-based approach. For the kmeans clustering approach, the user is required to provide the expected number of clusters of his/her model. For these two approaches, results are displayed in a table format in which each cluster is mapped to its related proteins.

Finally, we ran GOSS-FEAT, taking as the target set a list of 18 proteins annotated to GO:0051409 in order to identify the most statistically relevant biological processes in which these proteins are involved. We used the GO-universal metric, Wang *et al* and Resnik approaches and results are shown in Table 3. Once again, these results depend on the semantic similarity measure used and looking at these results, only the GO-universal approach was able to output the GO term used to identify proteins used as the target set, namely *response to nitrosative stress* GO:0051409. This application suggests that the GO-universal approach may constitute an effective solution to the GO metric problem for the next generation of functional similarity metrics [22].

**Table 3:** Running the GOSS-FEAT for specific GO Ids and using different GO term semantic similarity approaches

**Approach**	**GO-ID**	**GO name**	**Level**	**Reference fuzzy**	**Target fuzzy**	**p-value**	**Corrected**
				**Frequency**	**Frequency**		**p-value**
GO-Universal	GO:0051409	Response to nitrosative stress	3	91	18	0.00e+00	0.00e+00
	GO:0006979	Response to oxidative stress	3	90	18	8.08e-13	5.17e-11
	GO:0052572	Response to host immune response	7	92	8	1.16e-07	7.45e-06
Wang et al.	GO:0006979	Response to oxidative stress	3	226	18	0.00e+00	0.00e+00
	GO:0046677	Response to antibiotic	4	164	18	0.00e+00	0.00e+00
	GO:0001666	Response to hypoxia	5	163	18	0.00e+00	0.00e+00
	GO:0006974	Response to DNA damage stimulus	5	419	18	0.00e+00	0.00e+00
	GO:0009432	SOS response	5	316	18	0.00e+00	0.00e+00
	GO:0034605	Cellular response to heat	5	351	18	0.00e+00	0.00e+00
	GO:0071500	Cellular response to nitrosative stress	5	365	18	0.00e+00	0.00e+00
	GO:0075136	Response to host	5	293	12	9.10e-08	5.82e-06
Resnik	GO:0009432	SOS response	5	294	18	0.00e+00	0.00e+00
	GO:0034605	Cellular response to heat	5	296	18	0.00e+00	0.00e+00
	GO:0071500	Cellular response to nitrosative stress	5	294	18	0.00e+00	0.00e+00
	GO:0009267	Cellular response to starvation	6	294	18	0.00e+00	0.00e+00
	GO:0071456	Cellular response to hypoxia	7	370	18	0.00e+00	0.00e+00
	GO:0071732	Cellular response to nitric oxide	7	369	18	0.00e+00	0.00e+00
	GO:0006284	Base-excision repair	8	361	18	0.00e+00	0.00e+00
	GO:0006289	Nucleotide-excision repair	8	361	18	0.00e+00	0.00e+00
	GO:0006307	DNA dealkylation involved in DNA repair	9	424	18	0.00e+00	0.00e+00
	GO:0052059	Evasion or tolerance by symbiont of host-	11	319	18	1.63e-12	1.04e-10
		produced reactive oxygen species					
	GO:0052060	Evasion or tolerance by symbiont of host-	11	319	18	1.63e-12	1.04e-10
		produced nitric oxide					
	GO:0051701	Interaction with host	4	35	6	1.05e-07	6.74e-06

### Other GO semantic similarity tools and DaGO-Fun

As mentioned previously, there have been numerous tools developed for producing GO term and protein semantic similarity scores. These include web interfaces and software tools very often implemented in the R programming language. These tools, together with functional similarity measures they support, are shown in Table 4. As pointed out previously, each approach performs differently for different applications. For example, the maximum approach achieves good performance for prediction of protein-protein interactions compared to other approaches [24]. The best-match average approaches perform better in protein function prediction and validation [9], and protein or gene clustering, while the average approach is good for detecting similar protein sequences from their GO annotations [1]. The existing tools allow researchers to browse the specific approaches separately for their proteins of interest, but an integrated tool for exploring all the IC-based similarity approaches to allow researchers to choose the most relevant approach for their applications did not exist previously. DaGO-Fun solves this by allowing researchers to browse the integrated set of all IC-based GO semantic similarity approaches. The similarity scores produced are scaled (normalized) to enable comparison between different approaches, and in the future we will work on enabling multiple options to be run, with a summary or merging of results where possible.

**Table 4:** IC-based GO semantic similarity tools and functional similarity measures (FSM) they support

		**GO-semantic similarity features implemented**		
**Tool**	**Format**	**Family**	**Approach**	**FSM**
G-SESAME	Web	Topology-based	Wang et al.	ABM
		Annotation-based	Classical Resnik, Lin	Average
			and Jiang & Conrath	
ProteInOn	Web	Annotation-based	Classical Resnik, Lin	BMA and
			and Jiang & Conrath	SimGIC
			GraSM	
FuSSiMeg	Web	Annotation-based	Classical Resnik, Lin	Max
			and Jiang & Conrath	
			GraSM	
FunSimMat	Web	Annotation-based	Classical Resnik, Lin	ABM
			and Jiang & Conrath	
			SimRel (enhancement)	
SemSim	R	Topology-based	Wang et al. Method	ABM
		Annotation-based	Classical Resnik, Lin	Average
			and Jiang & Conrath	
			SimRel (enhancement)	
csbl.go	R	Annotation-based	Classical Resnik, Lin	SimGIC
			and Jiang & Conrath	Average
			GraSM and SimRel	

In terms of input size, the G-SESAME and FuSSiMeg web tools accept only one pair of GO terms or proteins. The ProteInOn tool may take up to 1000 GO terms or proteins according to its authors, for which the tool outputs all pairs of similarity scores, and the FunSimMat tool has unlimited input size. We aim to let the DaGO-Fun tool calculate results for as many user inputs as possible, however, because of limitations in computational resources, we have to balance the maximum number of GO terms, and GO term and protein pairs for each user query. Thus, the DaGO-Fun tool accepts up to 5000 GO terms when retrieving GO term IC scores, in which case the tool will display only 10 of them per page, but all GO term features can be retrieved by downloading them in a text file. For GO term semantic similarity scores as well as for protein functional similarity scores, the user can enter at most 3000 pairs. Entries beyond the maximum limitations will be ignored. Unfortunately if you have cases where your data exceeds these limitations, it is necessary to divide the input data, run the DaGO-Fun tool separately, and merge the results at the end of the process. Alternatively you can contact the authors who are willing to collaborate and run large data sets for analysis.

## Conclusions

We have developed the DaGO-Fun tool, a customized web-based GO semantic similarity resource. This userfriendly online interface produces GO term information content (IC), GO term semantic similarity and protein functional similarity scores, which may assist experimental and computational biologists in several applications involving protein analyses at the functional level. These include gene list enrichment, protein function prediction and comparison, clustering genes or proteins based on their GO annotation information, and ranking disease candidate proteins or identification of novel disease candidate proteins. This tool will be updated quarterly (every three months) using an automated scheme in order to remain up to date to meet requirements of ever increasing applications in the biomedical field. The DaGO-Fun tool is freely available, meaning that one is free to copy, distribute, display and make unrestricted non-commercial use of it under the GNU General Public Licence provided that it is done with appropriate citation of the tool and its components.

Despite the wide range of IC-based GO semantic similarity applications and the existence of several approaches to meet requirements of these applications, there was no tool available that integrates all these IC-based approaches. Thus, researchers had to implement these approaches themselves, use different tools for different approaches, or download the individual software packages, making extraction and comparison of these scores difficult and time-consuming. The DaGO-Fun tool overcomes these issues, providing easy retrieval of IC-based GO term semantic similarity and protein functional similarity scores within a large protein annotation dataset from GOA-UniProtKB. It ensures that GO semantic similarity data are conveniently accessible to researchers and can effectively be used to investigate functional similarity between proteins based on their GO annotations. In addition, we implemented some biological applications of these semantic similarity measures, including protein classification and identification based on their GO annotations, and term enrichment analysis.

Future work includes facilitating the search for functional similarity between sets of GO terms. In this case, the user will have to provide pairs of sets of GO terms using a specified key linking the sets. This will undoubtedly improve the flexibility of the DaGO-Fun tool, by allowing users to produce functional similarity scores for their own predicted set of genes given their GO annotations. We will assess the relevance of two IC-term based functional similarity approaches introduced here, namely SimDIC and SimUIC and evaluate the use of annotation-based functional similarity approaches in the context of the GO term IC topology-based family. Finally, we will be expanding the DaGO-Fun tool to include some other applications of GO semantic similarity in protein analyses, such as protein function prediction, annotation system comparisons, and disease protein prioritization.

## Availability and requirements

DaGO-Fun is available at http://web.cbio.uct.ac.za/ITGOM, accessible by any user with a standard web browser but has only been tested on Mozilla Firefox 20.0. The whole system is implemented using a LAMP (Linux-Apache-MySQL and PHP/Python) platform. This means that the DaGO-Fun tool is implemented under free software (GNU General Public Licence) using a Linux Apache server with a database structured in a relational model using MySQL version 14.14 Distrib 5.5.31, and the web interface is implemented in PHP version 5.3.10 and standard HTML. The back-end is composed of a set of query processing programs implemented in Python version 2.7.3.

## Competing interests

The authors declare that they have no competing interests.

## Authors’ contributions

NJM generated and supervised the project, and finalized the manuscript. GKM designed and implemented the tool, and wrote the manuscript. Both authors read and approved the final manuscript. NJM approved the production of this paper.
